# Editorial: AI applications for diagnosis of breast cancer

**DOI:** 10.3389/frai.2023.1247261

**Published:** 2023-10-17

**Authors:** L. J. Muhammad, Alessandro Bria

**Affiliations:** ^1^Department of Information Technology, Bayero University, Kano, Kano State, Nigeria; ^2^Department of Electrical and Information Engineering, University of Cassino, Cassino, Italy

**Keywords:** Artificial Intelligence, breast cancer, machine learning, diagnosis, prediction

Artificial Intelligence (AI) has recently gained significant attention and broader recognition due to its vital role in various sectors, most notably the health sector (Alsayed et al., 2021; Algehyne et al., 2022). In the health sector, AI is predominantly employed for the diagnosis and prognosis of numerous fatal diseases, specifically breast cancer (AlShourbaji et al., 2022), COVID-19 (Sarumi and Aouedi, 2021), and Coronary Artery Disease (Muhammad and Algehyne, 2021), among others. Therefore, we initiated a unique research call for a Research Topic titled “AI Applications for the Diagnosis of Breast Cancer” at Frontiers in Artificial Intelligence under the Medicine and Public Health Section of the Journal. The aim of this study was to enhance the early diagnosis and screening of breast cancer through the use of novel AI-based methods, techniques, and frameworks developed by a global array of medical consultants, researchers, and oncologists. Consequently, this topic managed to accumulate various AI-based novel contributions from several scholars. These novel contributions comprise AI-based methodological and practical frameworks and improved methods, approaches, and tools that provide accurate and efficient breast cancer diagnosis. We received 45 manuscripts, each undergoing meticulous reviews by field experts. Of the 45 manuscripts, only 21 were accepted and published after multiple revisions based on the reviewers' and editors' comments.

In the inaugural paper on the Research Topic (Sun et al.), selected preoperative clinical features (ultrasound features and stress elastography) were utilized to construct an AI-based model that refines the assessment of benign and malignant breast lumps. The model achieved a specificity of 0.92, surpassing other models. Thus, this model has the potential to enhance preoperative detection of benign and malignant breast tumors and reduce unnecessary breast biopsies.

Lee et al. developed a machine learning support vector machine (SVM) trained with radiomic features derived from breast magnetic resonance imaging (MRI) to predict improvements in centrally diagnosed ductal carcinoma *in situ* (DCIS) following surgical resection through needle biopsy (CNB). The experimental results illustrate that this approach attains remarkable accuracy and serves as a useful tool for predicting histological improvements in DCIS.

Gong et al. performed a meta-analysis evaluating the diagnostic power of radiomics for predicting axillary lymph node metastasis (ALNM) and sentinel lymph node metastasis (SLNM) in breast cancer. The meta-analysis included thirty studies involving 5,611 patients, and the results demonstrated that radiomics exhibits exceptional diagnostic performance in predicting ALNM and SLNM in breast cancer.

Bansal et al. assessed Thermalytix, a novel machine learning technique to enhance breast thermography, as an adjunct in breast disease assessment. The study results display acceptable sensitivity and specificity in the use of mammography, establishing it as the standard across the patient population and particularly in women with dense breast tissue.

Zhang et al. a multi-parameter MRI model incorporating ensemble learning to explore the predictive performance of ALNM in preoperatively invasive breast cancer. The results of this study demonstrated that this model can provide clinicians with extensive preoperative information about axillary breast cancer, assisting in accurate clinical decision-making.

Xu et al. developed a radiograph based on grayscale ultrasound (USA) for the preoperative prediction of lymphatic vessel invasion (LVI) in patients with pathologically confirmed invasive breast carcinoma (IDC) T1. The results showed satisfactory preoperative prediction of LVI in pT1 IDC patients.

Recently, circulating tumor DNAs (ctDNAs) have become a focus for scientists in relation to personalized treatment, early detection, diagnosis, and accurate prognosis of breast cancer. Consequently, Ji et al. performed a bibliometric analysis on research trends related to tumor DNA associated with breast cancer from 2012 to 2021. This study explored the value of ctDNA in breast cancer using bibliographic analysis, providing a comprehensive and intuitive understanding of the topic and revealing research trends over the past decade.

Dihge et al. developed an artificial neural network algorithm for non-invasive lymph node staging as a decision support system to facilitate the potential omission of axillary surgery in breast cancer patients at low risk of lymph node metastasis. The algorithm provides healthcare decision support and facilitates the preoperative identification of patients who may be good candidates to avoid surgical staging of the axillary region.

Ding et al. identified a novel immunosuppressive tumor microenvironment subtype of triple-negative breast cancer with high PD-L1 expression and potential resistance to ICH treatment. These results provide novel insights for understanding the molecular mechanisms underlying resistance to ICH therapy and for developing suitable immunotherapeutic strategies for patients with distinct molecular characteristics.

Chen et al. proposed a deep learning model based on C2FTrans to diagnose breast masses using mammographic density. The model could readily distinguish between benign and malignant mass lesions in the breast on mammography images, potentially serving as an auxiliary diagnostic tool for radiologists in the future.

Liu et al. developed a learning-based EfficientNetV2 model for identifying and diagnosing malignant structural distortions in mammography. The model improves the overall accuracy of mammography in detecting malignant structural distortions.

The measurement of the quality of life (QOL) of patients with breast cancer assists in assessing the therapeutic efficacy of medical interventions and informs clinical decisions. To this end, Zhou et al. examined the QOL of breast cancer patients using both traditional and modern anchor-based methods to determine a minimal clinically important difference (MCID). When assessing the QOL of patients with breast cancer, MCID values calculated using different methods were found to be relatively similar. Anchor-based methods render the results of the MCID more clinically interpretable by introducing clinical variables. Clinicians and researchers can select the appropriate method according to their research preferences.

Zarean Shahraki et al. predicted a time-related survival probability for breast cancer patients over 30 years of age with various molecular subtypes, who were diagnosed with invasive breast cancer between 1991 and 2021. In this retrospective analysis of 3,580 patients, a random forest learning algorithm identified tumor status, age at diagnosis, and lymph node status as the best set of variables for predicting breast cancer survival probability.

Xu et al. developed an Integrated Clinical Radiological model (ICR) to investigate the feasibility of predicting progression-free survival (PFS) in patients with breast cancer using a positron emission tomography/18F-fluorodeoxyglucose computed tomography (FDG PET/CT) radiographic signature and clinical parameters. The model independently predicted PFS in patients with breast cancer, outperforming both the clinical model alone and the RAD index.

Wu et al. proposed a novel multiregion dose-gradient-based multistacking classifier framework incorporating Bayesian optimisation parameter tuning for accurate prediction of symptomatic RD2+ in patients with breast cancer undergoing radiotherapy. This approach represents a substantial improvement over conventional models, allowing early identification of patients at risk of radiation-induced toxicity and providing a highly accurate prediction of symptomatic RD2+ in patients with breast cancer.

Murtas et al. developed a clinical radiographic model based on features extracted from digital breast tomosynthesis (DBT) images and clinical factors that could aid in differentiating between benign and malignant breast lesions. The study's results suggest that this model can be considered a support tool for radiologists in the diagnosis of breast tumors, particularly during initial screenings.

Sun et al. investigated the utility of radiometric features based on T1-weighted imaging (T1WI), T2-weighted imaging (T2WI), and ADC maps in identifying TP53 mutations in triple-negative breast cancer. The results showed that radiometric features combined with a Support Vector Machine learning algorithm can achieve better predictive performance than other conventional models.

Saha et al. carried out a natural language processing (NLP) scope assessment of radiological reports related to breast cancer. The study's results showed that NLP applications can automate routine tasks, allowing physicians to focus on more complex cases or tasks, and enable consistent quality control measures for routine mammograms and breast cancer treatment.

Wang et al. evaluated the capability of ultrasound imaging-based integrated radiomics to differentiate between fibroadenomas (FAs) and pure mucinous carcinomas (P-MCs). The study retrospectively enrolled 170 patients with either FA or P-MC (120 in the training group and 50 in the testing group) with clear pathological confirmation. Four hundred and sixty-four radiomic features were extracted from conventional ultrasound images (CUS), and radiomics scores (RadScores) were constructed. The results indicated that the Clin + CUS + RadScore model could effectively differentiate between FA and P-MC, thus enhancing radiologists' confidence and supporting clinical decision-making.

Sultana et al. investigated the potential role of serum cfDNA levels as an early diagnostic marker for breast cancer using UV spectroscopy, fluorescence, and real-time qPCR assays. They proposed a sequential combination of NanoDrop, Quantus, and RT-qPCR methods for the preliminary assessment of total circulating cfDNA, which is both cost-effective and comprehensive. The findings of the study demonstrated that RT-qPCR combined with fluorescence measurement could identify a statistically significant difference in cfDNA levels between patients with breast cancer and healthy controls.

To et al. proposed an automated method leveraging the power of deep learning techniques. In this method, the DUV-WSI image was divided into small patches, and features were extracted using a pretrained CNN. A gradient boosting tree was then trained on these features for patch-level classification. An ensemble learning method combined patch-level classification results and regional importance to determine the margin status. The proposed method outperformed standard deep learning classification methods on DUV breast surgery samples. It can be used to enhance classification performance and more effectively identify cancerous areas.

The Research Topic contributed immensely for development of the novel frameworks, methods, approaches, and tools that provide efficient diagnosis of breast cancer.

## Author contributions

All authors listed have made a substantial, direct, and intellectual contribution to the work and approved it for publication.
